# Nutritional Ingredients Modulate Adipokine Secretion and Inflammation in Human Primary Adipocytes

**DOI:** 10.3390/nu7020865

**Published:** 2015-01-26

**Authors:** Tania Romacho, Philipp Glosse, Isabel Richter, Manuela Elsen, Marieke H. Schoemaker, Eric A. van Tol, Jürgen Eckel

**Affiliations:** 1 Paul Langerhans Group for Integrative Physiology, German Diabetes Center, Auf’m Hennekamp 65, 40225 Düsseldorf, Germany; E-Mails: tania.romacho@ddz.uni-duesseldorf.de (T.R.); Philipp_Glosse@gmx.de (P.G.); richter_isabel@gmx.de (I.R.); manuela.elsen@ddz.uni-duesseldorf.de (M.E.); 2 Global Discovery, Mead Johnson Pediatric Nutrition Institute, Middenkampweg 2, Nijmegen, 6545 CJ, The Netherlands; E-Mails: Marieke.Schoemaker@mjn.com (M.H.S.); Ric.VanTol@mjn.com (E.A.T.)

**Keywords:** milk proteins, casein hydrolysate, EPA, DHA, ARA, LC-PUFAs, adiponectin, adipocytes

## Abstract

Nutritional factors such as casein hydrolysates and long chain polyunsaturated fatty acids have been proposed to exert beneficial metabolic effects. We aimed to investigate how a casein hydrolysate (eCH) and long chain polyunsaturated fatty acids could affect human primary adipocyte function *in vitro*. Incubation conditions with the different nutritional factors were validated by assessing cell vitality with lactate dehydrogenase (LDH) release and neutral red incorporation. Intracellular triglyceride content was assessed with Oil Red O staining. The effect of eCH, a non-peptidic amino acid mixture (AA), and long-chain polyunsaturated fatty acids (LC-PUFAs) on adiponectin and leptin secretion was determined by enzyme-linked immunosorbent assay (ELISA). Intracellular adiponectin expression and nuclear factor-κB (NF-κB) activation were analyzed by Western blot, while monocyte chemoattractant protein-1 (MCP-1) release was explored by ELISA. The eCH concentration dependently increased adiponectin secretion in human primary adipocytes through its intrinsic peptide bioactivity, since the non-peptidic mixture, AA, could not mimic eCH’s effects on adiponectin secretion. Eicosapentaenoic acid (EPA), docosahexaenoic acid (DHA), and DHA combined with arachidonic acid (ARA) upregulated adiponectin secretion. However, only DHA and DHA/ARA exerted a potentanti-inflammatory effect reflected by prevention of tumor necrosis factor-α (TNF-α) induced NF-κB activation and MCP-1 secretion in human adipocytes. eCH and DHA alone or in combination with ARA, may hold the key for nutritional programming through their anti-inflammatory action to prevent diseases with low-grade chronic inflammation such as obesity or diabetes.

## 1. Introduction

The prevalence of childhood obesity has dramatically increased in recent years [1]. It has been proposed that rapid weight gain during the first years of life is related to the development of obesity later in life [2]. Early nutrition plays a crucial role in the pathogenesis of diseases later in adult life, particularly metabolic diseases [3,4]. Therefore, the impact of nutritional factors on the development of obesity and its related metabolic complications later in adult life has generated considerable interest in the field of infant nutrition [5,6].

Adipose tissue (AT) is a major endocrine organ with a pivotal role in the onset of metabolic diseases by means of adipokines [7]. In obesity, AT enlargement triggers an imbalance in adipokine secretion. In these diseases the circulating levels of several pro-inflammatory adipokines, such as leptin and TNF-α, are enhanced, followed by a lower expression of adiponectin, which contributes to the characteristic low-grade chronic inflammatory status [8,9,10]. AT inflammation represents a potential key pathogenic link between obesity, insulin resistance, and type 2 diabetes [7,11].

Human milk provides important nutritional factors and is a complex mixture of growth factors, cytokines, hormones, adipokines, and other proteins and lipids [12]. Human milk not only contains intact proteins but also casein peptides with potential biological activity. Since enzyme hydrolysis is used to reduce the allergenicity of milk protein or generate bioactive or tolerogenic sequences, some infant formulas contain extensive casein hydrolysates (eCH). Extensive hydrolysis predominantly produces smaller peptides (95% lower than 1 kDa). It is suggested that complimentary to the hypoallergenic moiety, eCH have been proposed to exert anti-inflammatory effects and anti-obesity effects [13]. Breast milk is also a main source of the long chain polyunsaturated fatty acids (LC-PUFAs) eicosapentaenoic acid (EPA, 20:5n-3), docosahexaenoic acid (DHA; 22:6n-3) and arachidonic acid (ARA; 22:4n-6). DHA and ARA are found in breast milk at a 1:1.47–2 ratio [14]. Nowadays infant formulas provide LC-PUFAs, more specifically ARA and DHA, as these two essential fatty acids cannot be sufficiently synthesized and are crucial for the child’s overall development. Early life supplementation with DHA/ARA improved cognitive and visual capacities in children [15]. Several studies have proposed that the intake of certain n-3 series LC-PUFAs may exert beneficial effects in the prevention of obesity and its related complications, both in rodents and humans [16,17,18,19,20,21]. Importantly, recent data suggest that changes in the balance of essential LC-PUFAs may critically determine adipose tissue development during infancy and program the body for later obesity onset in adulthood [22]. In this context, PUFAs of the n-6 series such as linoleic acid and ARA can stimulate adipogenesis through the activation of the nuclear receptors peroxisome proliferator-activated receptor (PPAR) -γ and δ. On the other hand, dietary n-3 LC-PUFA supplementation has been proven capable of reducing body fat and preventing or even reversing insulin resistance in rodents [23]. n-3 LC-PUFAs like EPA and DHA display anti-inflammatory properties, increase fatty acid oxidation, and prevent adipocyte hypertrophy in rodents [24]. Animal studies also show that DHA/ARA supplementation early in life reduces body weight gain and adiposity later in life [25]. The mechanism by which LC-PUFAs affect body weight gain and adipogenesis are largely unknown. However, there is limited information available regarding the effects of LC-PUFAs on human adipocyte function and the impact of these fatty acids on the inflammatory status.

Using a unique cellular model of human primary adipocytes, the impact of the extensive casein hydrolysate eCH, AA, (a non-peptide amino acid mixture), and LC-PUFAs on adipokine secretion and inflammatory status was studied.

## 2. Materials and Methods

### 2.1. Materials

Cell culture plastic ware was purchased from Sarstedt (Nümbrecht, Germany). Reagents for SDS-PAGE were supplied by Amersham Pharmacia Biotech (Braunschweig, Germany) and by Sigma (Munich, Germany). Collagenase NB4 was obtained from Serva (Heidelberg, Germany). Complete protease inhibitor cocktail tablets were from Roche (Mannheim, Germany). Fetal calf serum was supplied by Gibco (Invitrogen, Carlsbad, CA, USA). The eCH and AA were provided by Mead Johnson (MJN, Evansville, IN, USA). Unless stated otherwise elsewhere, all other chemicals were purchased from Sigma.

### 2.2. Preparation and Handling of the Nutritional Factors

#### 2.2.1. Profile of the Extensive Casein Hydrolysate (eCH)

**Figure 1:**
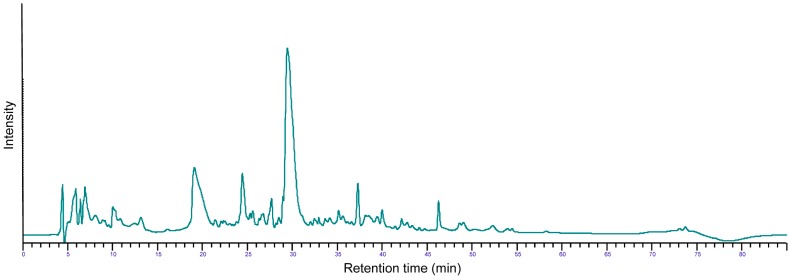
RP-HPLC chromatogram of the extensively hydrolyzed casein displaying its peptide profile.

Analytical reversed-phase high performance liquid chromatography (RP-HPLC) of extensively hydrolyzed casein was performed according to Visser *et al.* [26]. In brief, samples were dissolved in 2% acetonitrile and 0.1% trifluoroacetic acid. Detection was carried out by UV-absorption at 220 nm. The peptide profile as seen from the RP-HPLC is presented in Figure 1.

Peptide lengths and their relative abundance were determined by automated Edman degradation and amino acid determination as described previously [26]. The extensive casein hydrolysate (eCH) consists of a mixture of peptides with a length distribution shown in Table 1, with a large proportion of smaller sequences of less than 1000 Da.

Based on this hydrolysate, a non-peptide amino acid mixture, similar to the one present in the hydrolysate, was used. A stock solution (5% (w/v)) of the milk fraction or the AA was prepared in α-MEM medium, supplemented with an antibiotic anti-mycotic solution at 0.2% (Gibco). Aliquots were stored at −20 °C until further use.

**Table 1:** Peptide length distribution of the extensively hydrolyzed casein (eCH) as assessed by Edman degradation. Peptide lengths and their relative abundance are presented, showing a mixture of peptides with a large proportion of smaller sequences of less than 1000 Da, typical of an extensive hydrolysate.

Peptide length	Relative signal (%)
1–2	44.0
2–3	17.4
3–4	9.7
4–5	7.9
5–6	5.2
6–7	4.5
7–8	3.0
8–9	1.9
9–10	1.7
10–11	1.1
11–12	1.1
12–13	0.9
13–14	0.5
14–15	0.5
15–16	0.6

#### 2.2.2. Preparation and Handling of LC-PUFAs

Stock solutions (30 mM) of the LC-PUFAs were prepared in sterile DMSO at 1% O_2_ inside a hypoxia chamber (BioSpherix) in order to prevent oxidative degradation of the fatty acids. The stock solutions of the LC-PUFAs were protected from light and stored in the dark at −80 °C until further use. Sodium salts of oleic acid (18:1 cis-9, OA) were dissolved in sterile deionized water to create a 164.5 mM stock solution. Prior to treatment the fatty acids were complexed with bovine serum albumin (BSA) in αMEM as previously described by us in a 2.5 mM fatty acid per 1 mM BSA ratio [27]. The adipocytes were treated for 24 h with 50 or 100 μM of EPA, DHA, OA or 100 μM of ARA, combined with 50 μM of DHA. BSA with DMSO (at a final concentration of 0.001%) served as the vehicle control.

### 2.3. Isolation and Culture of Primary Human Preadipocytes and Experimental Design

Subcutaneous adipose tissue was obtained from healthy lean and moderately overweight women undergoing plastic surgery (*n =* 45, BMI = 27.83 ± 4.37, 47.18 ± 10.94 years). The procedure was approved by the ethical committee of the Heinrich Heine University (Düsseldorf, Germany). Human primary adipocytes were isolated by collagenase digestion, as previously described by our group [28]. Isolated cell pellets were resuspended in Dulbecco’s Modified Eagle Medium (DMEM)/F12 medium supplemented with 10% FCS, seeded in six-well or 12-well culture dishes, respectively, and maintained in an incubator at 37 °C with 5% CO_2_. After reaching confluence (day 0 of differentiation), cell cultures were incubated in an adipocyte differentiation medium (DMEM/F12, 33 mmol/L biotin, 17 mmol/L D-panthothenic-acid, 66 nM insulin, 1 nM triiodo-L-thyronine, 100 nM cortisol, 10 mg/mL apo-transferrin, 50 mg/mL gentamycin, 0.25 mg/mL amphotericyn B, 15 mmol/L HEPES, 14 nmol/L NaHCO3, pH 7.4) with troglitazone (5 µM) only for the first 3 days. Once differentiation was started, the cells were further incubated in adipocyte differentiation medium for a total differentiation period of 14 days. The medium was changed every 2–3 days. Differentiated adipocytes were characterized by Oil Red O staining and immunofluorescence with a specific anti-perilipin-1 antibody. 14 days after induction of differentiation, adipocytes were treated for 24 h with the milk fractions (eCH or AA) at 3 different concentrations (0.001%, 0.1%, and 1% w/v) or LC-PUFAs (at 50 µM or 100 µM), alone or in combination with eCH for 24 h. All the treatments were performed in α-modified minimum essential medium (αMEM, Gibco).

### 2.4. LDH Activity

In order to validate the appropriate incubation conditions, incubation with the eCH and the AA mixture was evaluated in the adipocytes. Fully differentiated adipocytes were exposed to the fractions dissolved in medium at several concentrations (0.01%, 0.1%, and 1%). After 24 h of incubation, the cell supernatants were collected and processed to measure lactate dehydrogenase (LDH) activity with the colorimetric LDH cytotoxicity detection kit (Roche) following the manufacturer’s instructions.

### 2.5. Neutral Red Staining

For determination of cell viability, the cells were seeded in 12-well cell culture plates and challenged with eCH, AA (0.01%, 0.1%, and 1%), or EPA, ARA and DHA (100 μM) for 24 h. After the treatment, the supernatants were discarded and the cells were exposed to a sterile neutral red solution for 3 h in a humidified incubator at 37 °C (5% CO_2_). The neutral red solution was carefully removed by aspiration and cells were washed twice with PBS. The neutral red staining was extracted from the cells with an elution medium (50% (v/v) ethanol and 1% (v/v) acetic acid) under gently shaking on an orbital shaker for 10 min at RT. Eluted neutral red was quantified in the elution medium and the absorbance was measured at 540 nm wavelength with an Infinite M200 microplate reader (Tecan, Männerdorf, Germany).

### 2.6. Oil Red O Staining

After 24 h incubation with the different milk fractions at 1% at day 14 of differentiation, the adipocytes were stained with Oil Red O in order to assess lipid accumulation. After washing with PBS, the adipocytes were incubated with a fixative solution consisting of 71% picric acid (v/v), 24% acetic acid (v/v), and 5% formaldehyde (w/v). The fixative was removed and cells were washed three times with PBS. Lipids were stained by incubation for 10 min in a 60% isopropanol solution with 0.3% Oil Red O. The Oil Red O dye was eluted from the cells with pure isopropanol and the absorbance was measured at 500 nm wavelength with a microplate reader.

### 2.7. Western Blot

Cells were treated as indicated and scrapped in lysis buffer (20 mM MOPS, 2 mM EGTA, 5 mM EDTA, 1 mM dithiothreitol, 1% Triton X-100, complete protease inhibitor cocktail and PhosStop phosphatase inhibitor cocktail, pH 7.0). After shaking for 2 h at 4 °C, the lysates were centrifuged at 10,000 g for 15 min. The protein concentration in the lysates was determined by the Bradford method (Bio-Rad, Hercules, CA, USA). 10 μg of protein were separated by SDS–PAGE using 10% horizontal gels and transferred to polyvinylidene fluoride membranes (Millipore, Billerica, MA, USA) in a semidry blotting system. Membranes were blocked with 5% non-fat dry milk or BSA in TBS containing 0.1% Tween for 1 h at RT and incubated with the respective primary antibodies overnight at 4 °C. After washing, membranes were incubated with the corresponding HRP-coupled secondary antibody and processed for enhanced chemiluminescence detection using an Immobilon HRP substrate (Millipore, Billerica, MA, USA). Signals were visualized and quantified with a VersaDoc 4000 MP workstation (BioRad). Antibodies were used as follows: anti-adiponectin (Abcam, Cambridge, UK) diluted 1:1000, anti-phospho-NF-κB (P-p65) (Ser536), (Cell Signaling Technology, Frankfurt, Germany), and anti-β-actin (Abcam, Cambridge, UK).

### 2.8. Adiponectin, Leptin, and Monocyte Chemoattractant Protein-1 (MCP-1) Enzyme-Linked Immunosorbent Assay (ELISA)

The effect of the casein hydrolysate and the LC-PUFAs alone or in combination with the casein hydrolysate on adiponectin and leptin release was analyzed. The isolated human preadipocytes were carefully counted and the same cell number per well was plated. After the differentiation period, the cells were treated with the eCH (0.01%, 0.1%, and 1%) and the LC-PUFAs (50 μM or 100 μM) alone or in combination for 24 h. The supernatants were collected and stored at −20 °C for analysis of the respective adipokine content.

Adiponectin, leptin, and MCP-1 expression in the supernatants was monitored by ELISA using specific kits purchased from BioVendor GmbH (Heidelberg, Germany), R & D systems (Cambridge, UK) and Diaclone (Besançon Cedex, France) respectively. The respective assays were performed following the manufacturer’s instructions.

### 2.9. Data Analysis

Results are expressed as mean ± SEM. Statistical analysis was performed using Student’s *t* test and one-way ANOVA, followed by the Bonferroni *post hoc* test or Dunnett’s test to determine statistical significances. All statistical analyses were performed using Prism (GraphPad, La Jolla, CA, USA), considering a value of *p* < 0.05 as statistically significant.

## 3. Results

### 3.1. eCH and AA Do Not Alter Cell Viability or Morphology

**Figure 2:**
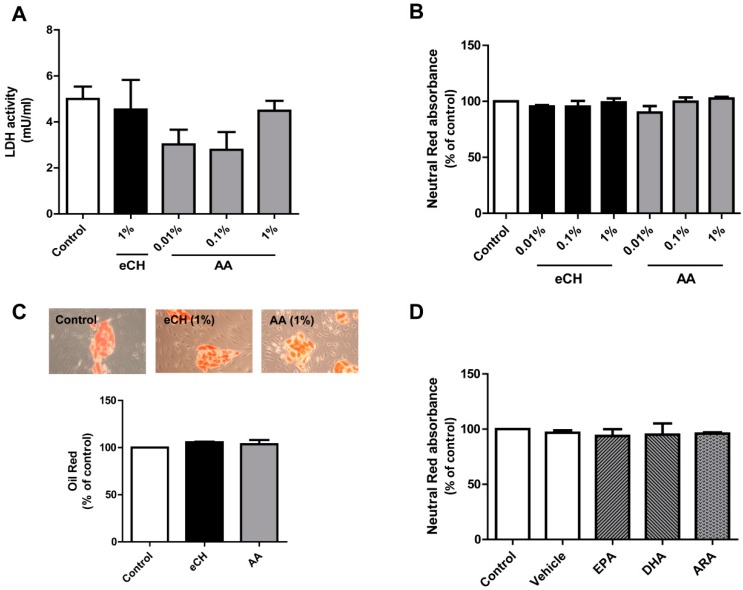
Validation of incubation conditions of nutritional factors. (**A**) LDH activity in the supernatants of adipocytes exposed for 24 h to eCH and AA (at 0.01%, 0.1% or 1%). Data are shown as the mean ± SEM of 5 independent experiments. (**B**) Neutral red incorporation (expressed as % of control levels) in the adipocytes exposed for 24 h to eCH and AA (at 0.01%, 0.1% or 1%). Data are shown as the mean ± SEM of 5 independent experiments. (**C**) Intracellular triglyceride content assessed by Oil Red O staining (expressed as % of control levels) of adipocytes untreated (control) or challenged with eCH or AA, respectively (at 1%). Data are shown as the mean ± SEM of 3 independent experiments. Representative brightfield microphotographs of every treatment at a magnification of 200X are shown on top. (**D**) Neutral red incorporation (expressed as % of control levels) in the adipocytes exposed for 24 h to EPA, DHA, or ARA (100 μM). Data are shown as the mean ± SEM of 3 independent experiments.

In order to validate the incubation conditions in our cell system, we explored the effect of the casein hydrolysate eCH and a non-peptide amino acid mixture (AA) (at 0.01%, 0.1%, and 1%) on parameters of cell cytotoxicity such as lactate dehydrogenase (LDH, L-lactate:NAD oxidoreductase, EC. 1.1.1.27). LDH is a ubiquitous enzyme found in the cytoplasm of all cells and tissues in the animal body that is released upon cytotoxic cellular stress. Since intrinsic LDH activity has been previously described in bovine milk [29], we first aimed to determine the intrinsic LDH activity in eCH and AA. We discarded an intrinsic effect from the milk components since the absorbance values for eCH and AA at 1% were below the detection range. The LDH activity in the supernatants of the adipocytes stimulated with eCH and AA did not differ significantly from the LDH levels detected in the supernatants of the untreated cells. The eCH and the AA fraction (1%) yielded a LDH activity of 4.54 ± 1.29 mU/mL and 2.97 ± 6.77 mU/mL, respectively, while the basal LDH activity was 4.997 ± 0.54 (Figure 2A). To discard any potential detrimental effect of eCH, AA or the LC-PUFAs and the respective vehicle on cell vitality, we additionally explored the effects of a 24 h challenge with these nutritional factors with a highly sensitive method, neutral red incorporation in the cells. As observed in Figure 2B, neither eCH nor AA altered neutral red incorporation in the cells. Thus, at the highest concentration eCH and AA respectively triggered a neutral red absorbance of 99.09 ± 3.59 and 102.60 ± 1.27% of control. We further analyzed in more detail the effect of eCH and AA at 1% on triglyceride accumulation (Figure 2C). The triglyceride content was spectrophotometrically analyzed with Oil Red O staining. Triglyceride content in the untreated adipocytes (control) did not significantly differ from the cells challenged for 24 h with eCH and AA at 1% (105.7 ± 0.61 and 103.6 ± 4.48, respectively). It can be observed in the microphotographs that the cell morphology was unaltered in all the treatments (Figure 2C).

Since DHA exposure (at 200 μM for 24 h) has been reported to promote apoptosis in mouse 3T3-L1 adipocytes [30], we wanted to further validate our incubation conditions in order to discard potential negative side effects on the cells or cellular stress-related adiponectin release. As observed in Figure 2D, neither the vehicle nor any of the LC-PUFAs impaired cell vitality.

### 3.2. eCH Upregulates Adiponectin Secretion in a Concentration-Dependent Manner

**Figure 3:**
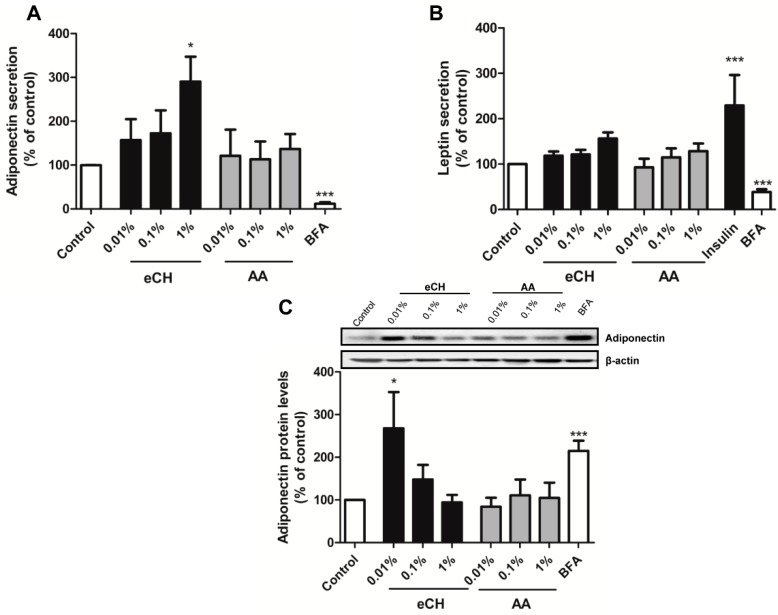
The eCH fraction upregulates adiponectin secretion without increasing intracellular levels. Effect of 24 h challenge with the eCH or AA fractions (at 0.01%, 0.1% and 1%) on adiponectin (**A**) or leptin secretion (**B**). Results were expressed as % of adiponectin/leptin levels triggered by the vehicle. Data are shown as the mean ± SEM of 3–9 independent experiments. Brefeldin A (1 μg/mL) was used as an inhibitor of protein secretion, while insulin (50 mM) was employed as a positive control for leptin secretion. (**C**) Effect of 24h challenge with the eCH or AA fractions (at 0.01%, 0.1% and 1%) on adiponectin intracellular protein levels. Results were expressed as % of adiponectin basal levels. Data are shown as the mean ± SEM of 3–4 independent experiments. * *p* < 0.05, *** *p* < 0.001 *vs.* % of untreated control.3.3. EPA and DHA Upregulate Adiponectin Secretion.

Once the *in vitro* setting for incubating the milk components was validated, we compared the effect of 24 h exposure of the cells to the eCH and the amino acid mixture AA on adipokine secretion. The eCH fraction upregulated adiponectin secretion in a concentration-dependent manner. This effect was independent of the amino acid content in the eCH, since the AA fraction alone did not alter adiponectin levels (Figure 3A). Although a concentration-dependent trend was observed, neither the eCH fraction nor the amino acid mixture significantly upregulated leptin secretion (Figure 3B).

In order to determine if the effect of eCH exerted on adiponectin secretion was due to an increase of *de novo* synthesis of the adipokines, we explored the effect of eCH on intracellular adiponectin expression by Western blot (Figure 3C). We included the AA fraction as a control to prove the specificity of the effect promoted by eCH. Interestingly, at 0.01% the eCH fraction significantly upregulated adiponectin protein levels, which gradually decreased with increasing concentrations of the fraction. Analogously to adiponectin secretion, the amino acid mixture did not affect intracellular adiponectin levels.

### 3.3. EPA and DHA Upregulate Adiponectin Secretion

The potential differential effects of n-3 and n-6 LC-PUFAs on adiponectin and leptin secretion were explored in human primary adipocytes; the cells were challenged for 24 h (at day 14 of differentiation) with either 50 or 100 μM of LC-PUFAs—EPA, DHA, and ARA—coupled to BSA. 5-aminoimidazole-4-carboxamide-1-β-riboside (AICAR; 1 mM) was used as a positive control for adiponectin secretion, whereas brefeldin A (BFA; 1 μg/mL), an inhibitor of the endoplasmic reticulum—Golgi transport, served as a negative control.

**Figure 4:**
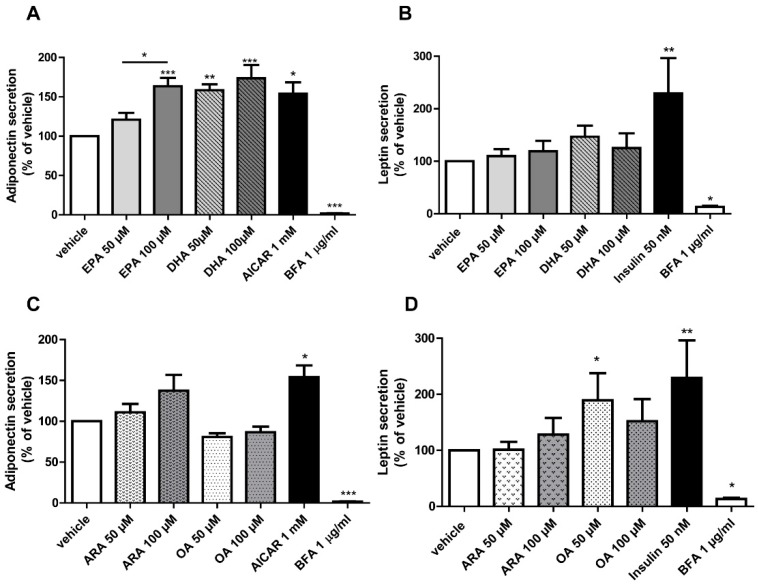
EPA and DHA differentially upregulate adiponectin secretion in human adipocytes. Effect of EPA and DHA incubation (50 and 100 μM) for 24 h on adiponectin (**A**) or leptin secretion (**B**) in the adipocytes. Effect of ARA and oleic acid (OA) (50 and 100 μM) for 24 h on adiponectin (**C**) or leptin secretion (**D**). Adiponectin and leptin concentration in the supernatants was determined by ELISA. AICAR (1 mM) was used as a positive control for adiponectin secretion and insulin (50 nM) was used as a positive control for leptin secretion, while brefeldin A (1 μg/mL) was used as a negative control. Results were expressed as % of adiponectin/leptin levels triggered by the vehicle. Data are shown as mean ± SEM of 3–5 independent experiments. * *p <* 0.05, ** *p <* 0.01, *** *p <* 0.001 s *vs.* vehicle.

Incubation of adipocytes with EPA at 100 μM significantly increased the concentration of secreted adiponectin compared to the vehicle and EPA at 50 μM (Figure 4A). Adiponectin levels in the supernatants were also significantly enhanced both by DHA at 50 μM (158.40% ± 7.50% of vehicle; * *p <* 0.01) and 100 μM (173.60% ± 16.87% of vehicle; *** p < 0.001), respectively (Figure 4A). Thus, the n-3 LC-PUFAs EPA and DHA increased adiponectin secretion to a similar extent as AICAR (Figure 4A).

Analogously to the studies on adiponectin secretion, secreted leptin protein was determined in the supernatants of adipocytes challenged with LC-PUFAs with a specific ELISA kit. Regarding the n-3 LC-PUFAs, 24 h incubation with EPA or DHA did not exert any significant effect on leptin secretion compared to the vehicle control (Figure 4B).

The n-6 PUFA ARA did not significantly enhance adiponectin or leptin secretion (Figure 4C,D, respectively). On the contrary, the most prominent effect on leptin secretion was observed with the monounsaturated fatty acid OA (50 μM), included to validate the specificity of the effects of PUFAs. At this concentration, OA provoked a significant increase in leptin secretion of approximately two-fold compared to the vehicle (189.30% ± 48.58% of vehicle; *p <* 0.05) (Figure 4D). Interestingly, OA at 100 μM did not display a significant upregulation of leptin secretion, although at this concentration secreted leptin levels were approximately 52% higher compared to the vehicle (151.90% ± 39.73% of vehicle) (Figure 4D). ARA did not elicit a significant effect on leptin secretion in the adipocytes.

In order to explore if the n-3/n-6 LC-PUFAs upregulated intracellular adiponectin content, the protein levels of this adipokine were analyzed after 24 h exposure to ARA, DHA or EPA alone (at 50 or 100 μM, respectively). We observed no significant alteration of adiponectin levels after the 24 h incubation period (data not shown).

**Figure 5:**
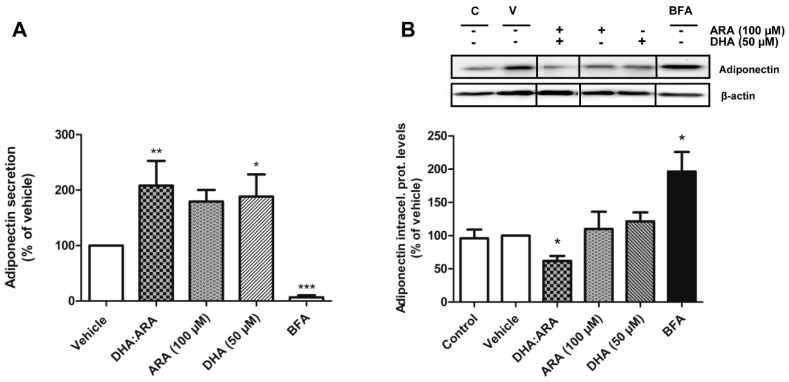
DHA and DHA/ARA upregulate adiponectin secretion in human adipocytes without affecting intracellular protein levels. Effect of the DHA/ARA combination (50 μM:100 μM), ARA (100 μM), or DHA alone (50 μM) on adiponectin secretion (**A**) or expression (**B**). Brefeldin A (1 μg/mL) was used as a negative control. Results were expressed as % of corresponding adipokine levels triggered by the vehicle. Data presented as mean ± SEM of 3–6 independent experiments. * p < 0.05; *** p < 0.001 *vs.* vehicle. (V: vehicle control, C: untreated control). A representative blot is shown on top.

DHA is often used in a LC-PUFA blend with ARA in a 1:1.47–2 ratio for formula application. Thus, we further aimed to determine if this n-3/n-6 combination could modulate adiponectin synthesis and secretion. The DHA/ARA combination significantly upregulated adiponectin secretion (Figure 5A). Regarding adiponectin intracellular levels, the LC-PUFAs combination dramatically reduced adiponectin protein levels inside the adipocytes (62.03% ± 7.24% of vehicle control levels), probably due to the increased secretion induced by the combination at 24 h exposure time (Figure 5B).

### 3.4. The DHA/ARA Combination Exerts a Prominent Anti-Inflammatory Effect

**Figure 6:**
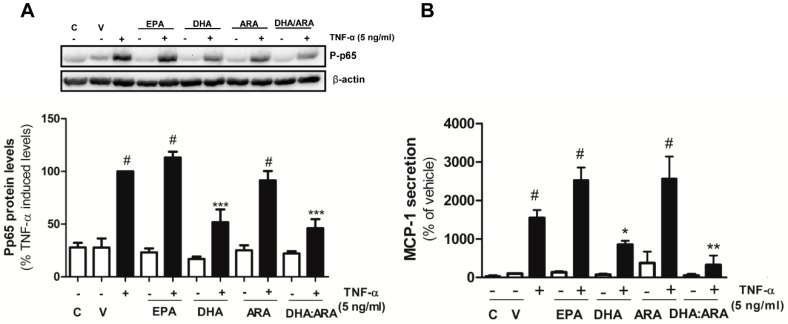
DHA and DHA/ARA prevent TNF-α-induced inflammation in human adipocytes. Effect of EPA, DHA, ARA (100 µM), and DHA/ARA (50 µM:100 µM) over 30 min pre-incubation on TNF-α-induced NF-κB activation for 24 h. (**A**) Results were expressed as % of P-p65 levels induced by TNF-α. Data presented as mean ± SEM of 3–9 independent experiments; (**B**) Effect of EPA, DHA, ARA (100 µM) and DHA/ARA (50 µM:100 µM) during 30 min pre-incubation on TNF-α induced MCP-1 secretion for 24 h by the adipocytes. Results were expressed as % of MCP-1 secretion levels induced by the vehicle. Data presented as mean ± SEM of 3–6 independent experiments. ^#^ *p <* 0.05 *vs.* vehicle control; * p < 0.05; ** p < 0.01; *** p < 0.001 *vs.* NF-κB activation levels induced by TNF-α. A representative blot is shown on top.

We next explored if the different LC-PUFAs—namely EPA, DHA, ARA and the DHA/ARA combination—could prevent TNF-α mediated NF-κB activation. The adipocytes were preincubated for 30 min with EPA, DHA, ARA (100 μM) and the DHA/ARA (50 μM:100 μM) prior to 24 h stimulation with TNF-α (5 ng/mL). As expected, TNF-α significantly upregulated the phosphorylated levels of the NF-κB subunit p65, indicating NF-κB activation. However, neither EPA nor ARA prevented the effect elicited by TNF-α. Importantly, the n-3 PUFA DHA, alone and in combination with ARA, significantly reduced NF-κB activation compared to TNF-α alone (51.81 ± 12.07 and 46.02 ± 8.69% of TNF-α—induced Pp65 levels, respectively) (Figure 6A). In order to further determine if this potent anti-inflammatory effect exerted by DHA alone or with ARA translated to a reduction of downstream targets of NF-κB, we studied the effect of the pre-incubation with EPA, DHA, ARA and the DHA/ARA combination prior to TNF-α challenge on MCP-1, as a key mediator of macrophage recruitment in AT. Accordingly, DHA alone and in combination with ARA dramatically reduced MCP-1 secretion by the adipocytes (1.80- and 4.74-fold reduction, respectively, compared to TNF-α-induced levels) (Figure 6B). Indeed, the DHA/ARA combination was more potent than DHA alone.

### 3.5. eCH Upregulates Intracellular Adiponectin Expression in Combination with DHA

**Figure 7:**
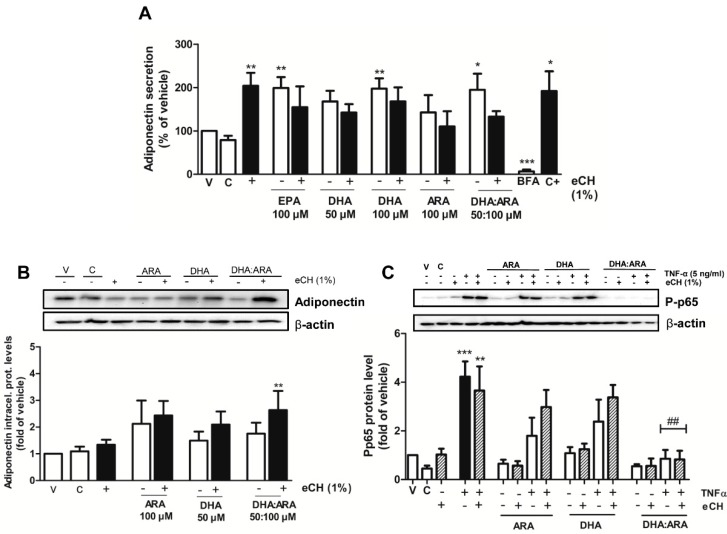
eCH does not potentiate the effect of n-3 LC-PUFAs on adiponectin secretion. (**A**) Effect of EPA (100 µM), DHA (50 and 100 µM), ARA (100 µM), and DHA/ARA (50 µM:100 µM), alone or in combination with eCH (1%), on adiponectin secretion. Results were expressed as % of adiponectin levels induced by the vehicle. Data presented as mean ± SEM of 4–8 independent experiments. (**B**) Effect of ARA (100 µM), DHA (50), and DHA/ARA (50 µM:100 µM) alone or in combination with eCH (1%) on adiponectin protein levels. Results were expressed as % of adiponectin levels induced by the vehicle. Data presented as mean ± SEM of 8–9 independent experiments. A representative blot is shown on top. (**C**) Effect of ARA (100 µM), DHA (50), and DHA/ARA (50 µM:100 µM), alone or in combination with eCH (at 1%), on TNF-α-induced NF-κB activation. Results were expressed as fold over vehicle of P-p65 levels. Data presented as mean ± SEM of 5–7 independent experiments. A representative blot is shown on top. * p < 0.05 vs. ** p < 0.01; *** p < 0.001 *vs.* vehicle control; ^##^ *p <* 0.01 *vs.* NF-κB activation levels induced by TNF-α. A representative blot is shown on top. (V: vehicle control, C: untreated control, C+: rosiglitazone 10 nM).

In order to determine if eCH could act synergistically with the LC-PUFAs and potentiate their previously observed effects on adiponectin secretion, the adipocytes were incubated for 24 h with eCH alone or in combination with EPA (100 μM), DHA (50 and 100 μM), ARA (100 μM), and the DHA/ARA combination (50 μM:100 μM). However, we did not observe a potentiation of eCH on the adiponectin secretion levels triggered by the LC-PUFAs (Figure 7A).

Interestingly, the eCH fraction significantly upregulated intracellular adiponectin levels when combined with DHA/ARA (Figure 7B). It is therefore likely that this effect is translated into an increased secretion at exposure times longer than 24 h.

Since a role has been proposed for casein hydrolysates as alleviators of inflammation [31,32,33], we next explored the potential anti-inflammatory effect of preincubation with the eCH alone or in combination with the potent anti-inflammatory combination of ARA and DHA, prior to TNF-α challenge. Although a trend towards reduction of inflammation was observed, the eCH fraction alone did not significantly reverse the effect exerted by TNF-α on NF-κB activation. However, the eCH fraction could not potentiate the anti-inflammatory effect exerted by the DHA/ARA combination, which completely blunted TNF-α induced phosphorylation of p65 (Figure 7C).

## 4. Discussion

In our study we aimed to explore the modulation of two adipokines, adiponectin and leptin, with a prominent role in metabolic programming. Adiponectin reaches its peak at birth and high levels are maintained during the neonatal period until they fall progressively during infancy [34]. Adiponectin circulating levels are decreased in the obese and insulin-resistant status, but are increased in normal weight individuals, thus representing a protective marker against obesity and type 2 diabetes [7]. There is substantial evidence from animal and clinical studies that certain dietary interventions can modulate adiponectin secretion in adults. However, there are scarce reports analyzing the evolution of adiponectin circulating levels in infants [35]. Importantly, high-molecular-weight adiponectin has been proposed as one factor explaining the reported divergence in weight between small for gestational age infants undergoing breastfeeding or formula-feeding [36].

We have identified eCH as a potent nutritional factor in terms of adiponectin secretion. A very recent study analyzing the proteomic profile of digested human breast milk in a simulated stomach model has identified α, β and κ casein in the milk *digestas* intermediate decay proteins [37], thus providing evidence for casein reaching the circulation. Furthermore, similar concentrations have been previously used in other *in vitro* cellular models to prove the anti-inflammatory and anti-proliferative effect of α, β and κ caseins, as well as casein hydrolysates [38,39,40,41].

In our study, eCH upregulated adiponectin release in a concentration-dependent manner, while the amino acid mix AA did not modify adiponectin basal release. These results are in line with the *in vivo* effects of eCH on autoimmune diabetes prevention. eCH intake from weaning until day 150 prevented the onset of type 1 diabetes in diabetes-prone BioBreeding rats, while the single amino acid mixture only delayed the onset of the disease [42]. This study suggests that there must be specific bioactive peptides present in eCH that make it much more active than the amino acid mixture AA alone. Furthermore, another group described that chronic intake of eCH reduced body mass gain and diet-induced obesity in mice [13]. In this line, dietary intervention with casein hydrolysates has been recently proposed to reduce both oxidative and nitrosative stress, as well as the expression of pro-inflammatory cytokines in the intestine and the islets of non-obese diabetic (NOD) mice [43]. However, in our *in vitro* setting we could not demonstrate the direct anti-inflammatory effect of eCH alone, since preincubation with eCH prior to challenge to TNF-α over 24 h was not sufficient to prevent NF-κB activation in the adipocytes.

Nevertheless, the capacity to upregulate adiponectin secretion in human adipocytes reported herein combined with the antioxidant properties reported for casein and casein-derived peptides make of this nutritional factor a potential promising candidate for nutritional programming [44]. However, in the context of nutritional programming, one limitation of our study is that the preadipocytes have been isolated from adult female donors. Thus, it would be extremely interesting to analyze in more depth the impact of casein hydrolysates and LC-PUFAs on preadipocytes isolated from children. Additionally, further research is needed to understand the active principles of this casein hydrolysate, e.g. using activity-guided fractionation.

The LC-PUFA concentrations chosen in our study (50 and 100 μM) are within the physiological range of n-3 LC-PUFA plasma concentrations reported after dietary ingestion of these factors [45], thus confirming the physiological relevance of the effects on adiponectin secretion exerted by EPA and DHA in our study. Furthermore, we demonstrated that these concentrations were neither toxic nor affected adipogenesis/triglyceride accumulation in the adipocytes.

The n-3 LC-PUFAs EPA (100 μM) and DHA (50 and 100 μM) significantly enhanced the secretion of adiponectin in human primary adipocytes. These results are in line with other *in vitro* studies using human and murine adipocytes [46,47]. In fact, Tishinsky *et al.* observed a significant increase in adiponectin secretion from a commercial line of human adipocytes treated for 24 h with 100 μM of EPA [47]. On the contrary, in this study, DHA at 100 μM only elicited a significant increase in secreted adiponectin after longer incubation times of, 36 and 48 h. Furthermore, it has recently been shown that 24 h incubation of murine 3T3-L1 adipocytes with either EPA or DHA at 125 μM significantly increased adiponectin release [46]. However, studies investigating the impact of n-3 LC-PUFAs on adiponectin secretion in rat visceral adipocytes and 3T3-L1 murine adipocytes provide conflicting results [19,46,48].

Our results support other *in vivo* studies demonstrating the beneficial effects of EPA and DHA on adiponectin secretion in both rodents and humans [20,23,49]. It has recently been suggested that the n-3 LC-PUFAs EPA and DHA act as natural ligands for PPARγ, and thereby modulate the synthesis and secretion of adiponectin *in vitro* [47,50]. It has been demonstrated that the PPARγ antagonist bisphenol adiglycidyl ether (BADGE) completely abolished DHA-induced adiponectin release in human adipocytes, while the inhibitor only partially attenuated the EPA-mediated increase in adiponectin secretion. The authors therefore proposed that DHA induces its stimulatory effect on adiponectin secretion exclusively in a PPARγ-dependent manner, whereas EPA likely acts via a combination of PPARγ activation and an unknown additional mechanism [47]. 

Since we observed an increase in adiponectin release that was not paralleled with an increase in adiponectin intracellular levels, the effect of EPA, DHA, and the DHA/ARA combination on adiponectin secretion may be not due to increased *de novo* synthesis. Since LC-PUFAs represent PPAR ligands, it is likely that the release of adiponectin is mediated by the release of readily recruited intracellular adiponectin pools. In this line, it is known that the PPARγ agonists, thiazolidinediones, selectively enhance the secretion of high molecular weight adiponectin through upregulation of the chaperone modulating adiponectin release Ero1-L alpha [51]. It has also been suggested that activation of the fuel-sensing enzyme adenine monophosphate kinase (AMPK) could be a potential mechanism mediating the stimulatory effects of n-3 LC-PUFAs on adiponectin secretion [52]. However, in our experimental setting we did not observe AMPK activation by the n-3 LC-PUFAS (data not shown).

Regulation of leptin in rodents during pregnancy and lactation may have long-term effects on metabolism. However, the consequences of postnatal hyperleptinemia remain contradictory since it can both enhance and reduce predisposition to obesity in adult life [52]. In our experimental setting, none of the LC-PUFAs significantly upregulated leptin secretion, and only the monounsaturated fatty acid OA enhanced the release of this adipokine. Furthermore, the effect of EPA on leptin secretion also remains controversial. It has been reported that EPA can stimulate leptin basal and insulin-stimulated leptin secretion (not a focus of our study) and mRNA expression in murine 3T3-L1 cells and rat primary visceral adipocytes, respectively [45,53]. On the contrary, another study showed that EPA inhibited leptin secretion and expression in both humans and rats *in vivo* and *in vitro* in rat visceral AT [54].

Adipocyte-derived factors such as TNF-α are significantly increased in obesity and are good predictors of the development of type 2 diabetes [55,56]. Our study demonstrates that casein hydrolysates and DHA/ARA in combination exert an anti-inflammatory effect by upregulating adiponectin secretion. Furthermore, DHA alone or combined with ARA reduces TNF-α-elicited NF-κB activation, thus preventing the release of MCP-1, a strong promoter of macrophage recruitment in the AT. However, we are aware that our study has been performed in preadipocytes isolated from subcutaneous AT and thus care should be taken to extrapolate these results to visceral AT.

Furthermore, TNF-α is also on the basis of acute inflammatory preterm birth complications such as bronchopulmonary dysplasia (BPD), retinopathy of prematurity (ROP), and necrotizing enterocolitis (NEC) [57]. Therefore, early supplementation with casein hydrolysates and LC-PUFAs may offer a therapeutic strategy to prevent short-term acute inflammatory complications on the one hand and complications derived from chronic long-term inflammation in the adipose tissue leading to insulin resistance and type 2 diabetes on the other. In animal studies, DHA/ARA supplementation has been reported to reduce body weight gain, and reducing plasma levels of metabolic parameters such as cholesterol later in life diminishes obesity and improves adipose tissue quantity and quality [25]. On the contrary, a mixture of conjugated linoleic acid (CLA) and n-3 LC-PUFA decreased adiponectin circulating levels in rats, while upregulating adiponectin expression in the retroperitoneal and subcutaneous depots [58].

In high fat fed mice, DHA alleviated adipose tissue inflammation by downregulating the expression of MCP-1 [59]. This effect was due to the DHA-derived metabolite resolvin D1. We observed that DHA but not EPA inhibited TNF-α-induced NF-κB-mediated activation, resulting in reduced MCP-1 secretion by the adipocytes. Although both of the n-3 LC-PUFAs upregulated adiponectin secretion, only DHA exerted an anti-inflammatory effect in our experimental conditions. This differential effect may point toward a more efficient anti-inflammatory effect of DHA-derived resolvins compared to EPA-derived anti-inflammatory metabolites such as protectins.

Our group has previously demonstrated that globular adiponectin (gACRP) can directly modulate the secretory output of human primary adipocytes in an autocrine manner. Thus, adipocyte exposure to gACRP promoted a shift towards an anti-inflammatory profile reducing the release of inflammatory cytokines upregulated in obesity and contributing to insulin resistance, such as MCP-1, IL-6, or IL-8 [28]. Therefore, the anti-inflammatory effect exerted by DHA, alone or in combination with ARA, might be due to an increased release of adiponectin that can in turn prevent the effect of TNF-α on NF-κB activation and MCP-1 secretion in an auto/paracrine manner.

Several *in vitro* studies have shown the positive effects of adiponectin: decreasing inflammation, while improving insulin action and lipid oxidation. However, long-term adiponectin administration as a therapy for obesity and diabetes is not affordable due to high circulating adiponectin levels (in the range of μg/mL). Therefore, the potential of nutraceutical approaches such as administration of casein hydrolysates and n-3 LC-PUFAs to enhance adiponectin circulating levels are worth exploring further to determine if they may represent a useful and therapeutic strategy to fight and prevent obesity-related disorders.

Although EPA significantly upregulated adiponectin secretion, it did not exert an anti-inflammatory effect. The activation of the omega-3 fatty acid receptor G-protein-coupled receptor 120 (GPR120) by DHA (100 μM) markedly inhibited TNF-α-induced NF-κB activation in 3T3-L1 adipocytes [60]. It has been postulated that differences in the length and degree of saturation of these fatty acids may be critical determinants that affect the efficacy of n-3 LC-PUFAs’ interaction with GPR120 [61]. This may be the cause of the higher anti-inflammatory potency of DHA compared to EPA that we observed. Our results are in line with another study where chronic DHA pretreatment more potently inhibited LPS-induced NF-κB activity and cytokine expression in THP-1 macrophages compared to EPA [21].

n-3 PUFAS have been proposed to mediate the long term benefits of breast milk on obesity and diabetes. Most clinical trials performed in type 2 diabetic patients suggest that n-3 LC-PUFAs have no or reduced effects on metabolic control, while effectively reducing hypertriglyceridemia in these patients [23]. Since n-3 PUFAs cannot overcome diabetes once it is onset, early nutritional programming may hold the key to preventing obesity-related insulin resistance later in adult life. 

## 5. Conclusions

In summary, our data show that certain nutritional factors that can be found in human milk—specifically, casein-derived peptides and LC-PUFAs—can upregulate adiponectin secretion in human primary adipocytes. However, an anti-inflammatory autocrine effect of adiponectin was only observed when human primary adipocytes were challenged with DHA, alone or in combination with ARA. Future studies are needed to determine if the action of adiponectin, alone or combined with metabolites-derived from DHA/ARA, is responsible for the anti-inflammatory effect of the DHA/ARA combination on human adipocytes. DHA/ARA supplementation may represent an affordable and promising tool for preventing or alleviating AT inflammation. DHA, alone or in combination with ARA, may be useful in early nutritional programming by exerting a protective action against diseases characterized by low-grade chronic inflammation, such as obesity or diabetes.
